# Does the COVID-19 Pandemic Affect Morbidity and Mortality Rates of Emergency General Surgery? A Retrospective Study from a Single-Center Tertiary Greek Hospital

**DOI:** 10.3390/medicina57111185

**Published:** 2021-11-01

**Authors:** Eleni Karlafti, Emmanouil S. Benioudakis, Daniel Paramythiotis, Konstantinos Sapalidis, Georgia Kaiafa, Triantafyllos Didangelos, Antonios Michalopoulos, Isaak Kesisoglou, Christos Savopoulos

**Affiliations:** 1 Emergency Department, AHEPA University General Hospital of Thessaloniki, 55636 Thessaloniki, Greece; ikesis@auth.gr; 2 First Propeadeutic Internal Medicine Department, AHEPA University General Hospital of Thessaloniki, 55636 Thessaloniki, Greece; gdkaiafa@yahoo.gr (G.K.); didang@auth.gr (T.D.); chrisavopoulos@gmail.com (C.S.); 3 First Propaedeutic Surgery Department, AHEPA University General Hospital of Thessaloniki, 55636 Thessaloniki, Greece; danosprx@auth.gr (D.P.); amichal@auth.gr (A.M.); 4 Third Surgery Department, AHEPA University General Hospital of Thessaloniki, 55636 Thessaloniki, Greece; sapalidis@auth.gr

**Keywords:** emergency surgery, pandemic, COVID-19, SARS-CoV-2

## Abstract

*Background and Objectives*: The outbreak of the COVID-19 pandemic had a major impact on all aspects of health care. Few up-to-date studies have actually assessed the impact of COVID-19 on emergency surgeries. The aim of this study was to provide an overview of the impact of the pandemic relating to the emergency surgery performed, as well as morbidity and mortality rates during the first year of the pandemic (March 2020–February 2021) and during the control period. In this period, the first propaedeutic surgery department and the third surgery department of the University General Hospital of Thessaloniki “AHEPA” in Greece provided continuous emergency general surgery services. *Material and Methods*: The study is in a retrospective cohort and included patients who were admitted to the Emergency Department and underwent emergency general surgery during the control period (*n* = 456), March 2019–February 2020 and during the first year of the pandemic (*n =* 223), March 2020–February 2021. Gender, age, type of surgical operation (morbidity), ICU need, the patient’s outcome, and days of hospitalization were compared. *Results*: A total of 679 emergency surgeries were included. Statistically significant differences emerged between the two time periods in the total number of emergency surgeries performed (*p* < 0.001). The most common type of surgery in the control period was associated with soft tissue infection while, during the pandemic period, the most common type of surgery was associated with the hepatobiliary system. In addition, the mortality rates nearly doubled during the pandemic period (2.2% vs. 4%). Finally, the mean age of our sample was 50.6 ± 17.5 and the majority of the participants in both time periods were males. *Conclusions*: The COVID-19 pandemic changed significantly the total number of emergency general surgeries performed. Mortality rates doubled and morbidity rates were affected between the control and pandemic periods. Finally, age, gender, length of hospitalization, intensive care unit hospitalization, and laparoscopy use in patients undergoing emergency surgery during the pandemic were stable.

## 1. Introduction

On the 26th of February 2020, the first case of COVID-19 infection was confirmed at the “AHEPA” University General Hospital of Thessaloniki in Greece and on the 11th of March 2020, the World Health Organization declared the outbreak of COVID-19 as a pandemic [1]. The “AHEPA” University General Hospital was one of the first officially declared pandemic hospitals in Greece and has since treated patients suspected of having, or with, COVID-19, as well as patients without the COVID-19 infection. To this day, the first propaedeutic surgery department and the third surgery department of the “AHEPA” University General Hospital of Thessaloniki have continued to provide emergency general surgery services.

In the first months of the pandemic, the scientific community attempted to ascertain the disease process and its implications while the number of patients diagnosed with, or suspected of having COVID-19, increased [2]. However, the function of acute care surgery services had to continue, because any delay would raise the morbidity and mortality of the patients [3,4]. During the COVID-19 pandemic, a decrease and a delay were observed in even the most important surgical indications, such as acute appendicitis and testicular torsion [5,6,7,8]. Additionally, elective operations had to be postponed in order to mitigate the risk of new COVID-19 infections [9]. The decision-making process in emergency surgery, due to the lack of previous knowledge on COVID-19 management, was difficult. Therefore, after the first period of the pandemic, most surgical societies issued guidelines concerning emergency surgery, specifically in terms of patient and surgeon safety [10,11].

The COVID-19 pandemic required the medical community to overcome unknown features and to approach patients’ treatment in new ways [12]. The manner in which emergency surgeries were performed at the first propaedeutic surgery department and the third surgery department of the “AHEPA” University General Hospital had to be controlled and the level of prophylaxis needed to be at a maximum. The services provided for the patients suspected of, or diagnosed with, COVID-19 infection had to be optimal, while, at the same time, the spread of COVID-19 had to be limited. Moreover, the healthcare system had to protect the patients that were not infected with COVID-19 and to provide safe services. In parallel, the healthcare professionals were at high risk of exposure to COVID-19 and the performed surgeries had to be completed under increased precautions.

At present, there is minimal evidence addressing the impact of the COVID-19 pandemic on emergency surgery. The aim of this study was to provide an overview of the impact of the pandemic relating to the emergency surgery performed at the “AHEPA”University General Hospital during the first year of the pandemic (March 2020–February 2021) and to compare these data to equivalent data, one year before the pandemic (March 2019–February 2020). Moreover, we explored the morbidity and mortality rates of the emergency surgeries performed during the control and the pandemic periods.

## 2. Material and Methods

### 2.1. Data Collection

The electronic medical records of the patients included in this survey were collected from the first propaedeutic surgery department and the third surgery department of the “AHEPA” University General Hospital of Thessaloniki-Macedonia, Greece (a tertiary university hospital and referral center for COVID-19). Patients’ records were retrospectively reviewed in regard to their reason for presentation, age, gender, the need for hospital admission, COVID status, length of stay, admission to the intensive care unit, and in-hospital mortality. We compared two equal time periods, before the pandemic outbreak, from March 2019 until February 2020 (control period) and during the pandemic, from March 2020 until February 2021 (pandemic period). Only emergency surgeries were included in this study, as planned surgeries had stopped during the pandemic. Exclusion criteria were patients of all other medical procedures who did not require emergency surgery. The data were collected using a standardized data collection form and analyzed by the authors. Finally, a registration number was assigned to each patient to protect confidentiality.

### 2.2. Outcome Variables

The primary outcomes were the differentiation of mortality and morbidity rates, as well as the total number of surgeries occurring between the two time periods. The secondary outcome measures were the assessment of the differences between the surgeries’ morbidity, the age and gender of patients, admission to intensive care, and the result and the rates of laparoscopic surgeries occurring between the two time periods.

### 2.3. Statistical Analysis

Statistical analysis was performed using the statistical package SPSS, version 22 (SPSS Inc., Chicago, IL, USA). Normality of numerical data distribution was tested using the Kolmogorov–Smirnov test. Parameters are presented either as mean ± SD for continuous variables or frequencies (N) and percentages (%) for categorical variables. Independent Student’s *t*-tests for continuous variables and the Chi-square test for categorical variables, or the Fisher exact test with Monte-Carlo correction, were used to examine statistical differences between control and pandemic periods. Two-sided *p*-values < 0.05 were reported as significant.

## 3. Results

### Sociodemographic and Characteristics of the Surgeries during the Control and Pandemic Periods

**Figure 1:**
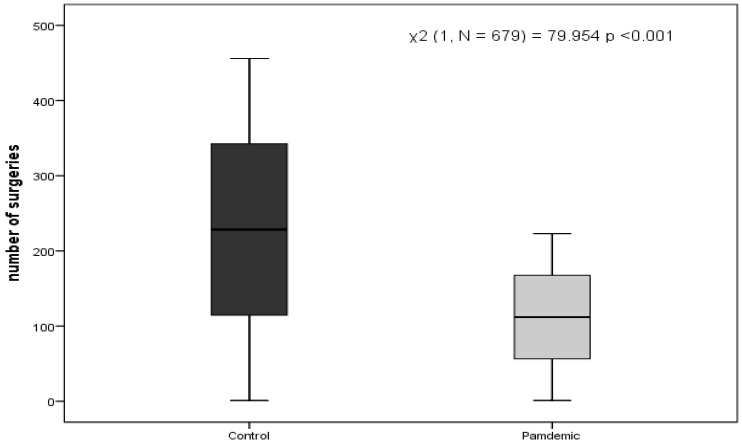
Box plot comparing the number of surgeries during the control and pandemic periods.

Six hundred and seventy-nine patients were included in the study. Four hundred and fifty six (67.2%) underwent surgery during the control period and 223 (32.8%) during the pandemic period, χ^2^ (1, N = 679) = 79.954 *p*<0.001, results are presented in the box-plot (Figure 1).

Three hundred seventy-six (55.4%) of the patients were males, 246 (65.4%) of whom underwent surgery during the control period and 130 (34.6%) during the pandemic period. In addition, 210 (69.3%) of female patients underwent surgery during the control period and 93 (29.7%) during the pandemic period. The mean age of our sample was 50.6 ± 17.5; for these data, the independent samples *t*-test between the control (Mdn = 52) and pandemic periods (Mdn = 50) was not significant, *p*-value of 0.171. Finally, mortality rates nearly doubled during the pandemic period (2.2% vs. 4%), although the total number was slightly lower in comparison with the control period. The results of the characteristics of the surgeries during the control and pandemic periods are presented in Table 1.

**Figure 2:**
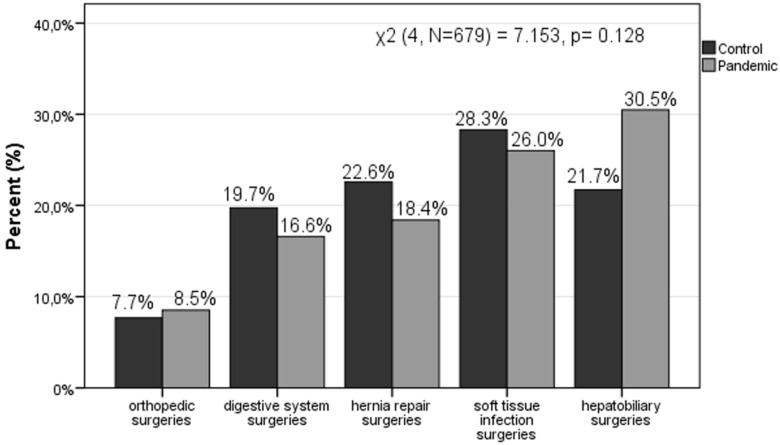
Bar-chart comparing the types of surgeries during the control and pandemic periods.

Additionally, we regrouped the different types of surgeries (morbidity) into five wide categories, to be able to further explore the differences. The five wide categories included orthopedic surgeries (fractures, dislocations and open wounds); digestive system surgeries (acute appendicitis, twist, abdominal and pelvic pain, malignant neoplasm of colon and unspecified ileus); hernia repair surgeries (umbilical hernia, inguinal hernia, abdominal hernia with gangrene); soft tissue infection surgeries (skin and rectal area abscesses); and finally hepatobiliary surgeries (abdominal and pelvic unspecified pain). The most common type of emergency surgery was fractures among the orthopedic surgeries; acute appendicitis among the digestive system surgeries; inguinal hernia among the hernia repair surgeries; and rectal area abscesses among the soft tissue infection surgeries and abdominal unspecified pain among the hepatobiliary surgeries. Morbidity was not significantly affected between the control and pandemic periods (χ^2^ (4, N = 679) = 7.153, *p* = 0.128). However, there were noticeable differences among the different types of surgeries that occurred between these time periods. The most common type of surgery that occurred during the control period was categorized as associated with soft tissue infection (28.3%), while during the pandemic period, the most common type of surgery that occurred was categorized as associated with hepatobiliary surgeries (30.5%). In addition, hernia repair surgeries were in a prominent position during the control period (22.6%), while soft tissue infection surgeries were in a prominent position during the pandemic period (26%). Results among the different types of surgeries during the control and pandemic periods are presented in the bar-chart (Figure 2). Finally, the mean days of hospitalization among the different types of surgeries during the control and pandemic periods are presented in Table 2.

## 4. Discussion

The results of the study prove beyond doubt the changing tendency in emergency general surgery during the COVID-19 pandemic. In our study, the total number of emergency surgeries during the first year of the pandemic showed a significant decrease, compared to the same period in March 2019–February 2020, one year before the pandemic. The phenomenon of “the disappearing of emergency surgery during the COVID-19 pandemic”, was described early, during the first months of the pandemic. Palisi et al. studied the data of the Surgical Emergency Department of their hospital in the first two months of the pandemic and noticed that the percentage of surgical accesses decreased significantly, compared with the same two-month period of the previous year [13]. The main cause of this reduction, according to the authors, was the fear of infection by the virus, especially among older people [13]. Further, Alimoglu et al. examined the number of consultations, hospitalization rates, and operations in their Surgery Clinic during the first two months of the pandemic and found them to have dramatically decreased when compared to the same time periods of 2018 and 2019 [14]. Moreover, a multicentre retrospective cohort study was performed, which included patients who underwent acute care surgery, in three tertiary care hospitals in Spain during a control period (11 March 2019 to 21 April 2019) and a pandemic period (16 March 2020 to 26 April 2020). This study highlighted a decrease in the daily number of acute care surgery procedures from 2.3 to 0.9 [15]. In addition, a prospective cohort study performed at a district general hospital in Scotland, which included all emergency general surgery patients, demonstrated a reduction (58.3%) in admissions as well as in the proportion of patients undergoing surgery, compared with the same time period in 2019 [16]. Thus, our results are consistent with previous studies which also highlighted a decrease in the number of emergency surgeries performed during the pandemic.

According to our study, there were no statistical differences in age, gender, length of hospitalization, intensive care unit hospitalization, and mortality of the patients between the two time periods. Relevant studies show the same data regarding gender [17,18]. In the same vein as our results, Dick et al. showed that, during the pandemic, patients who underwent emergency surgeries tended to be younger [16]. In accordance with our data, length of hospitalization was stable, as seen in the results of other studies [16,17,18], except that of Cano-Valderrama et al., which reported a reduction in hospitalization days during the pandemic [15], possibly due to the exertion of COVID-19 spread limitation, or even due to the need for an increase in bed capacity. In our study, the recorded deaths of patients undergoing emergency surgeries did not show a statistically significant difference between the two time periods and are in accordance with the data of relevant studies [15,19], although the general morbidity rate during the pandemic was higher [14,15]. Nevertheless, in our survey mortality rates nearly doubled during the pandemic period, although the number of deaths was slightly lower. This demonstrates the fact that mainly severe cases requiring emergency surgery were admitted to hospital. Additionally, this increase is based on the assumption that many patients delayed their care, thus resulting in an increase in the mortality rate due to the severity of the cases admitted to the hospital during the pandemic period [20]. Our results are in line with Lazati et al.’s nationwide analysis [5] and Dong et al.’s research [20].

Moreover, our study showed that the pandemic changed the morbidity of emergency general surgeries performed. More specifically, the number of digestive surgeries (19.7% vs. 16.4%), hernia repair surgeries (22.6 vs. 18.6%), and of soft tissue infection surgeries (28.3% vs. 26%) decreased, while hepatobiliary surgeries (21.7% vs. 30.5%) and orthopedic surgeries (7.7% vs. 8.5%) increased.On the one hand, a main hypothesis that can explain the decrease in some types of surgery during the pandemic, such as soft tissue infection surgeries, is that patients delayed going to the surgery department to avoid possible COVID-19 infection [21,22,23]. This delay may translate into a more advanced level of disease [22,24]. In addition, some authors referred to the management of mild forms of diseases such as cholecystitis, diverticulitis, and appendicitis at home, in order to avoid hospitalization; this fact could explain the discrepancies in the types of emergency surgeries performed during the pandemic [19,25]. On the other hand, the increase in the number of orthopedic emergency surgeries that was reported in our study may be due to an increase in the number of reported falls from heights, stairs, ladders, etc. [26,27]. In addition, the reduction in the number of emergency hernia repair surgeries reported is in line with previous studies [28]. We must point out that we compared the percentages within the two time periods of the emergency surgeries performed. It should be noted that all surgeries performed (including the hernia repair surgeries) could not have been further postponed. Finally, the increase in the number of hepatobiliary surgeries is a possible side effect of the SARS-CoV-2 virus, which directly damages the intrahepatic biliary system [29], while the liver appears as a potential target for infection [30]. In our survey, the increase appeared in the total percentage of hepatobiliary surgeries performed during the pandemic period. Thus, the SARS-CoV-2 side effect hypothesis emerged, even though we had no information about the history of SARS-CoV-2 virus infection.

Furthermore, an encouraging piece of data from our study is that all patients who underwent emergency surgery had a negative test for COVID-19, not only at the time of admission, but also at the end of their hospitalization. This reveals the effectiveness of the measures that were taken against the spread of COVID-19 infection in the first propaedeutic surgery department and the third surgery department, although the “AHEPA” University General Hospital was dealing with the pandemic. The use of personal protective equipment and the isolation of the emergency surgery services from COVID-19 services, while ensuring minimum contact between healthcare staff and patients, were the means that protected the patients from COVID-19 infection, although in the same hospital, there were patients infected with COVID-19 [31]. During admission to the Emergency Surgical Department, patients were screened for COVID-19 infection and the results were released after 48–72 h. This delay compelled the surgeons to approach patients under the assumption that they were COVID-19 positive, and to proceed to emergency surgeries, with all protective means, in order to minimize the possibility of COVID-19 spread from an infected patient [32]. Nevertheless, the surgical decision to proceed with emergency surgery would not have been affected by the COVID-19 test result. However, some studies refer to the fact that the majority of emergency general surgical patients did not undergo COVID-19 testing, although there is a specific guidance, mainly due to concerns over sensitivity as well as the high cost of testing [16,33].

Our study revealed similar percentages of laparoscopies used in the emergency surgeries during the pandemic in comparison to the control period. In addition, during the first months of the pandemic, there was confusion about the use of laparoscopy [34,35]. The society guidelines about laparoscopy, issued due to the new conditions caused by the spread of COVID-19, supported the elective use of the laparoscopic method when the clinical benefit outweighed the viral transmission risk, and the safety of the surgical team was guaranteed [36,37,38,39,40,41,42]. Non-operative management was implemented when appropriate. The emergency surgeon’s decision-making process took into consideration the World Society of Emergency Surgery (WSES) guidelines, the WSES Jerusalem guidelines [43], and the British Intercollegiate General Surgery Guidance on COVID-19 [44], but prioritized the haemodynamic status, age, and comorbidities of the patient with the aim of providing personalized patient management.

### Study Limitations

The main limitation of this study was the retrospective design of the project, the lack of randomization, the single-center study, the number of patients, and that patients undergoing non-operative treatment could not be further studied. To avoid this limitation, data from both periods were collected using the same methodology. The main strengths of our paper were the length of the project period and that it was performed in the University General Hospital located in Greece, where the pandemic was especially severe.

## 5. Conclusions

The COVID-19 pandemic changed significantly the total number of emergency general surgeries that occurred. The general emergency surgeries morbidity differed during the pandemic and themortality rates nearly doubled. More specifically, the percentage of hepatobiliary surgeries and orthopedic surgeries increased, in contrast with soft tissue infection surgeries, hernia repair surgeries, and digestive system surgeries, which were reduced. The age, gender, length of hospitalization, intensive care unit hospitalization, and laparoscopy use in patients undergoing emergency surgery during the pandemic remained stable. Finally, we must point out that the observance of precautionary measures against COVID-19 spread is important for the safety of the patients.

## Figures and Tables

**Table 1:** Characteristics of the surgeriesduring the control and pandemic periods.

Variables	Surgeries during Control Period (N = 456)	Surgeries during Pandemic Period (N = 223)	*p*-Value ^a^
	Mean ± SD N (%)	Mean ± SD N (%)	
**Gender**			0.284
Male	246 (53.9)	130 (58.2)	
Female	210 (46.1)	93 (41.7)	
**Age (years)**	51.2 ± 17.6	49.3 ± 17.1	0.171
**Laparoscopic surgeries**			0.526
Yes	73 (16)	40 (17.9)	
No	383 (84)	183 (82.1)	
**Days of hospitalization**	4 ± 8.6	4.6 ± 10.3	0.451
**Intensive care unit**			0.114
Yes	28 (6.1)	16 (7.2)	
No	428 (93.9)	207 (92.8)	
**Result**			0.120
Improvement	435 (95.4)	204 (91.5)	
Death	10 (2.2)	9 (4)	
Stable	11 (2.4)	10 (4.5)	

^a^*p*-values obtained by Student’s *t*-test for two independent means, and χ^2^ test or Fisher exact test with Monte-Carlo correction.

**Table 2:** Days of hospitalization.

Types of Surgeries	Control Period Mean ± SD	Pandemic Period Mean ± SD
Orthopedic surgeries	8.97 ± 19.5	5.16 ± 13.8
Digestive system surgeries	4.53 ± 7.8	7.24 ± 11.8
Hernia repair surgeries	2.34 ± 2	5.39 ± 18.2
Soft tissue infection surgeries	2.98 ± 6.7	2.47 ± 1.9
Hepatobiliary surgeries	5.03 ± 8.9	4.41 ± 4.3

## Data Availability

All materials supporting reported results are available from Eleni Karlafti, e-mail: linakarlafti@hotmail.com, 1st Propaedeutic Internal Medicine Department of the “AHEPA” University General Hospital, Aristotle University of Thessaloniki, Greece.
